# Improving the Texturization of Pea Protein Through the Addition of a Mung Bean Protein Extract Solution and Optimizing the Moisture Content, Screw Speed, and Extrusion Temperature

**DOI:** 10.3390/foods14213750

**Published:** 2025-10-31

**Authors:** Zhe Cheng, Shunzhang Ma, Ruiling Shen, Jilin Dong, Yunlong Li

**Affiliations:** 1 China Institute of Functional Food of Shanxi, Shanxi Agricultural University, Taiyuan 030031, China; chengzhe31@163.com; 2 Key Laboratory of Geriatric Nutrition and Health (Beijing Technology and Business University), Ministry of Education, Beijing 100048, China; mashunzhang@163.com; 3 College of Food and Bioengineering, Zhengzhou University of Light Industry, Zhengzhou 450001, China; shenrl1967@163.com

**Keywords:** pea and mung bean protein, high-moisture extrusion, protein structure, texturization degree

## Abstract

This study explores the use of a homemade mung bean protein extract solution (MP) as the moisture source in high-moisture extrusion to produce pea–mung bean composite textured protein (PMP). Single-factor experiments assessed the effects of MP addition amount (30–70%), screw speed (140–220 rpm), and extrusion temperature (140–180 °C) on the textural, physicochemical, and structural properties, followed by optimization using response surface methodology (RSM). MP addition amounts between 50% and 60% promoted higher surface hydrophobicity, a higher disulfide bond content, more ordered secondary structures, and a higher intrinsic fluorescence, accompanied by improved water- and oil-holding capacities, bulk density, and texturization degree (*p* < 0.05). Screw speeds of 160–180 rpm enhanced texturization and texture via increased shear and reduced residence time, whereas higher extrusion temperatures darkened the color (Maillard browning) and reduced texturization and the bulk density. RSM found that the optimal conditions were 53% MP, 160 rpm, and 150 °C, yielding a theoretical maximum texturization degree of 1.55, which was experimentally validated (1.53 ± 0.02). These findings support MP as an effective green moisture source to tailor the structure and functionality of pea-based high-moisture extrudates. Future work will integrate calibrated SME, sensory evaluation, and application testing in meat-analog formats.

## 1. Introduction

High-moisture extrusion (HME), a modern technique for preparing plant-based meat analogs, has been widely used in processing plant-based products. Plant proteins such as soy [1,2,3], pea [4,5,6] and peanut [7,8] are commonly used in extrusion. However, extrudates derived from a single protein source may not fully satisfy consumer requirements. Combining protein blends with extrusion can enhance the nutritional and functional quality of plant-based products [9]. For example, Rolandelli et al. [10] improved the texture of pea-protein HME products by adding zein, which promoted stratification and fiber formation. Gaber et al. [11] reported that adding oat protein improved the texture of pea protein extrudates. Chan et al. [12] showed that blended pea proteins improved the cooking quality of textured vegetable protein. Pea protein is a promising alternative to soy owing to its processability, low allergenicity, and nutritional value [13].

Previous studies have consistently identified moisture content, screw speed, and extrusion temperature as key factors influencing product quality in HME. A high moisture content facilitates protein melt viscosification, reducing its apparent viscosity and enabling shear forces during cooling to elongate and align denatured molecules into layered fibrils. In contrast, insufficient moisture leads to incomplete hydration, limiting protein mobility and resulting in dense, poorly oriented aggregates [14,15]. Toldrà et al. [16] reported that extrusion temperatures in the range of 143–150 °C produce faba-bean-protein-based HME products with optimal techno-functional properties. Increasing the extrusion temperature (120–160 °C) initially causes partial unfolding of plant proteins, exposing hydrophobic regions and free cysteine groups. This reduces the activation energy required for subsequent disulfide bond exchange and non-covalent interactions. Similarly, Chan et al. [12] found that screw speeds of 350–450 rpm, in combination with feed moisture levels of 38–42% (dry basis), significantly affect hydration kinetics and structural integrity in pea-protein textured products. Higher screw speeds increase the specific mechanical energy and local shear rate, promoting shear thinning and facilitating directional alignment of protein aggregates, while strain hardening occurs at the die outlet. Zhang et al. [17] observed that, under 350 rpm conditions, peanut protein showed a 40% increase in β-sheet content and simultaneously formed high-molecular-weight aggregates via disulfide bond cross-linking. Lyu et al. [18] further demonstrated that die temperature and screw speed critically influence the expansion ratio, bulk density, and specific mechanical energy of soy-based textured proteins. However, extreme processing conditions may impair the water absorption capacity and texture. After alignment, cooling in the die section triggers the reformation of disulfide bonds, hydrogen bonds, and hydrophobic interactions, thereby stabilizing fibrous structures. Recent FTIR studies have confirmed that the number of both covalent and non-covalent bonds increases with specific mechanical energy (SME), showing a positive correlation with tensile strength and sensory chewiness [19]. Collectively, these findings systematically establish moisture content, screw speed, and extrusion temperature as essential process parameters in HME.

Pea protein is gradually becoming a promising alternative to soy protein [20] due to its low allergenicity, high nutritional value, and versatility. Heat treatment such as baking can induce different changes in the overall structure of allergens [21]. Mung bean protein is recognized as a high-quality plant-derived protein due to its excellent amino acid profile, which is particularly rich in proline, glutamic acid, arginine, leucine, and phenylalanine [22]. Its remarkable thermal stability, along with superior water- and oil-binding capacities, makes it highly suitable for thermally processed food applications [23]. Studies have demonstrated that pure pea protein exhibits limited extrudability and texturization potential, but these properties can be significantly improved by blending it with other proteins [24]. Additionally, Zhang et al. [17] utilized response surface methodology (RSM) to optimize extrusion parameters, incorporating 30% pea protein to produce high-moisture meat analogs with enhanced quality. Ge et al. [25] research showed that mung bean protein had obvious advantages compared with soy protein. Its foaming stability and water-holding capacity were better than soy protein. Traditional methods often rely on chemical reagents to extract legume proteins, whereas starch-rich legumes can be directly dispersed in water, allowing proteins to co-solve with starch granules, which can then be separated through fractionation [26], achieving a more green and environmentally friendly extraction [27].

However, how pea protein and mung bean protein interact and how these interactions affect extrudate quality remain unclear. Therefore, this study investigates the feasibility and performance of preparing extrudates from compounded pea and mung bean proteins and examines the effects of extrusion parameters to obtain a highly texturized, fiber-like protein.

## 2. Materials and Methods

### 2.1. Materials

Pea protein isolate (PPI) was obtained from Yuwang Ecological Food Co., Ltd. (Yucheng, China). The protein, lipid, and water contents of the PPI employed in this study were 82.10 ± 0.10 g/100 g, 6.05 ± 0.09 g/100 g, and 6.24 ± 0.12 g/100 g, respectively. Peeled mung beans were purchased from a local supermarket (Zhengzhou Dennis Department Store Co., Ltd., Zhengzhou, China).

### 2.2. Preparation of Mung Bean Protein Extract Solution

The peeled mung beans were homogenized (A25, Shanghai Ouhe Machinery Equipment Co., Ltd., Shanghai, China) with deionized water at a ratio of 1:15 (*w*/*v*) and sonicated for 20 min at 300 W (SB-1200DT, Ningbo Xinzhi Biotechnology Co., Ltd., Ningbo, China). The slurry was then homogenized for five 1 min cycles at 10,000 rpm. The sample was centrifuged (TGL-16MS, Shanghai Luxiangyi Centrifuge Instrument Co., Ltd., Shanghai, China) at 3500 rpm for 20 min to remove the precipitate (starch and other macromolecules); the supernatant was collected and centrifuged again under the same conditions. The resulting supernatant was the mung bean protein extract solution (MP). The soluble protein content of the MP reaches 680 mg/100 g. The protein content was determined using the bicinchoninic acid (BCA) method.

### 2.3. Extrusion Experiments and Sample Preparation

Extrusion was performed on an extruder (Process 11, Thermo Fisher Scientific Inc., Waltham, MA, USA) equipped with a screw with a diameter of 11 mm and a 40:1 L/D ratio. The barrel had seven heating zones set to 40, 50, 60, 70, 90, 140, and 140 °C; zones 6 and 7 were the extrusion zones and were set identically within 140–180 °C. The die temperature was 120–130 °C, the die diameter was 5 mm, and the screw speed ranged from 140 to 220 rpm.

MP (m/m) was used as the moisture source for the PPI (dry basis) to prepare feed mixtures with target moisture contents of 30, 40, 50, 60, and 70% (*w*/*w*). A kitchen mixer (KM-993, KONKA Group, Guangzhou, China) was used for mixing, and samples were equilibrated at 4 °C overnight. In single-factor trials, when varying one parameter, the others were held constant (50% MP, 180 rpm, and 140 °C). This study employed a single-factor experimental design, independently examining the effects of MP addition, screw speed, and extrusion temperature. Each experimental factor includes a control sample prepared under baseline conditions (50% MP, 180 rpm, and 140 °C). Due to inherent batch-to-batch variation in the extrusion process, these independently produced control samples had acceptable minor numerical differences. All statistical analyses were based on comparisons between each experimental group and its respective control within the same series. Once a steady state was reached (continuous homogeneous output), pea–mung bean composite textured protein (PMP) was collected. Fresh PMP was used for textural quality analysis and a portion was freeze-dried (SCIENTZ-10ND/A, Ningbo Xinzhi Freeze-Drying Equipment Co., Ltd., Ningbo, China). Because the torque sensor was not calibrated, specific mechanical energy (SME) could not be accurately determined. Future work will incorporate online torque measurements for SME quantification. To facilitate replication, the complete screw configuration is provided so that relative energy inputs can be inferred.

### 2.4. Textural Properties

Texture profile analysis (TPA) was performed following the method of R. Zhang et al. [28] with minor modifications using a texture analyzer (TA-XT Plus, Stable Micro Systems, London, UK) equipped with a P/36R probe. The samples were compressed twice to 30% strain at 1 mm/s with a 5 s interval. Hardness, gumminess, and chewiness were obtained from force–time curves. The samples were cut to approximately 2 × 2 × 2 cm using an A/MORS cutter. The cutting parameters were a trigger force of 5 g, shear depth of 60%, pretest speed of 2 mm/s, test speed of 1 mm/s, and post-test speed of 2 mm/s. Longitudinal (FL) and transverse (FT) shear forces were recorded, and texturization degree was calculated as FL/FT. The measurements were repeated 10 times.

### 2.5. Expansion Ratio and Bulk Density

The expansion ratio (ER) was defined as the ratio of extrudate diameter to die diameter [29]. The extrudate diameter was measured using digital vernier calipers and the average of 10 random measurements were used to calculated the PMP diameter.

Expansion Ratio = (Extruded Product Diameter)/(Die Diameter (5 mm))(1)

The bulk density (BD) was calculated from the extrudate mass and dimensions, which were measured using a digital caliper [30].

BD = (4 × m)/(π × d^2^ × L) (2)

where L is the extrudate length (cm), d is the diameter (cm), and m is the mass (g). BD is reported in g/cm^3^.

### 2.6. Color Analysis

The L*, a*, and b* values of the sample surface were measured using a colorimeter (CR-400, Konica Minolta, Tokyo, Japan). Five different locations on the sample surface were randomly selected for measurement and the average value was taken. The color difference value (ΔE) of the extrudate was calculated using Equation (3). A standard white plate was used (L* = 98.2, a* = −1.0, and b* = 1.1).

(3) $$\Delta E=\sqrt{{\left(\Delta L\right)}^{2}+{\left(\Delta a\right)}^{2}+{\left(\Delta b\right)}^{2}}$$

### 2.7. Water- and Oil-Holding Capacities (WHC and OHC)

Following the method of Mazaheri et al. [31] with modifications, freeze-dried samples were ground and sieved (100 mesh). The sample (1.5 g) was mixed with 30 mL of deionized water or refined soybean oil in a 50 mL centrifuge tube, vortexed for 10 min at room temperature, and centrifuged at 4000 rpm for 20 min. The supernatant was decanted. The WHC or OHC (g/g) was calculated as

WHC (OHC) (g/g) = (m_2_ − m_1_)/m (4)

where m is the mass of the sample (g); m_1_ is the sum of the mass of the sample and the mass of the centrifuge tube (g); and m_2_ is the mass of the centrifuge tube after removing the supernatant (g).

### 2.8. Determination of Sulfhydryl (SH) Group and Disulfide Bond (DB) Contents

The free SH (FSH), total SH (TSH), and DB contents were determined as described by Sun et al. [32] with some modifications. The PMP (15 mg) was dispersed in 5 mL of Tris–glycine buffer or Tris–glycine–urea buffer to measure the FSH or TSH content, respectively. The protein sample solution was prepared by stirring (RH basic 1, IKA group, Staufen, Germany) at room temperature for 1 h. It was then centrifuged at 10,000 rpm for 10 min at 4 °C. Following this, 20 μL of Ellman’s solution was added. After a 5 min incubation, the absorbance at 412 nm was measured using a Multimode microplate reader (Spark, Tecan Trading AG, Switzerland). The total SH (TSH) and free SH (FSH) contents and DB content were calculated according to the following formulas:

(5) $$\mathrm{TSH}\left(\mathrm{FSH}\right)\left(\mu \mathrm{mol}/g\right)=\frac{73.53\times A_{412}\times D}{C}$$

(6) $$DB\left(\mu \mathrm{mol}/g\right)=\frac{\mathrm{TSH}-\mathrm{FSH}}{2}$$

where A_412_ is the absorbance of the supernatant at 412 nm; D is the dilution factor (5.02 for FSH and 10 for TSH); and C is the concentration of protein dispersed in the different buffers (mg/mL).

### 2.9. Surface Hydrophobicity (H_0_)

The surface hydrophobicity was determined using the ANS fluorescence (8-aniline-1-naphthalene sulfonic acid) probe method [33]. The PMP was dissolved in phosphate-buffered saline (PBS; 0.01 M, pH 7.0) and diluted to 0.04–0.20 mg/mL. The 4 mL sample was fully mixed with 20 μL of an ANS solution (8 mM, pH 7.0) and reacted in the dark for 2 min. A 4 μL volume of the sample was mixed with 20 μL of ANS (8 mM, pH 7.0) and incubated in the dark for 2 min. Fluorescence was measured at an excitation wavelength of 390 nm and emission wavelength of 470 nm (F-4600; Hitachi High-Tech Corporation, Tokyo, Japan). The slope of fluorescence intensity versus protein concentration was taken as H_0_.

### 2.10. Scanning Electron Microscopy (SEM) Observations

The microstructure of the freeze-dried PMP was observed using a scanning electron microscope (Sigma300, Carl Zeiss AG, Oberkochen, Germany). The sample was glued with conductive adhesive tape and sprayed with gold for 1 min. The microstructure of the PMP was observed after scanning using the electron microscope at an accelerating voltage of 3 kV and 5000× magnification.

### 2.11. Fourier Transform Infrared Spectroscopy (FTIR)

Following the method of Liu and Hsieh [34], the freeze-dried sample powder and KBr were mixed at ratio of 1:100 (*w*/*w*), pressed into pellets (HY-12, Tianguang New Optical Instrument Technology Co., Ltd., Tianjin, China), and analyzed (TENSOR 27, Bruker, Billerica, USA). The conditions were as follows: resolution of 4 cm^−1^, 64 scans, and wavelength range of 4000–400 cm^−1^. The amide I region (1700–1600 cm^−1^) was curve-fitted using Peakfit 4.12 (SPSS Inc., Chicago, USA): First, the baseline-corrected and normalized TXT data were imported, the 1700–1600 cm^−1^ region was extracted, Fourier deconvolution was performed, and the peaks were automatically found. Then, the peak positions were manually set according to four regions: 1654–1660 cm^−1^ (α-helix), 1665–1680 cm^−1^ (β-sheet), 1680–1700 cm^−1^ (β-turn), and 1660–1665 cm^−1^ (random coil). The peaks were repeatedly refit until R^2^ remained constant. Finally, the percentage of each sub-peak area relative to the total amide I band area was recorded as the corresponding secondary structure content.

### 2.12. Intrinsic Tryptophan Fluorescence Spectroscopy

The PMP was dispersed in PBS (0.1 M, pH 7.0), and a diluent with a protein content of 0.2 mg/mL was prepared. A fluorescence spectrophotometer (F-4600, Hitachi High-Tech Corporation, Totyo, Japan) was used for testing. The test conditions were as follows: excitation wavelength of 280 nm; scanning range of 300–400 nm; and slit width of 5 nm.

### 2.13. Response Surface Experimental Design

The three extrusion parameters were optimized in the single-factor experiment and in the response surface experiment: MP addition amount (%, X_1_), screw speed (rpm, X_2_), and extrusion temperature (°C, X_3_). The experimental combinations were planned using response surface methodology. A total of 17 combinations, including 5 replications of the center point to calculate the error sum of squares, were tested using Design Expert software (version 13) and regression equations and a 3D graph were generated. The response value was the texturization degree of the samples. The relationships between the coded values of independent and dependent variables were established to fit the experimental data, as demonstrated below.

(7) $$Y=\beta_{0}+\sum_{i=1}^{3}\beta_{i}X_{i}+\sum_{i=1}^{3}\beta_{i}X_{i}^{2}+\sum_{i=1}^{2}\sum_{j=i+1}^{3}\beta i_{j}X_{i}X_{j}$$

where *Y* is the dependent variable; *β*_0_, *β_i_*, *β_ii_*, and *β_ij_* represent the regression coefficients for constant, linear, quadratic, and interactive effects, respectively; and *X_i_* and *X_j_* denote the independent variables. The effects of the factors on the response were expressed as surface and contour plots to visualize the relationship between the response and the independent variables and to acquire the optimal conditions for the process [35].

### 2.14. Statistical Analysis

All measurements were performed in at least triplicate and are reported as the mean ± standard deviation. Data visualization used OriginPro 2022 (OriginLab Corporation, Northampton, MA, USA). Statistical analyses were conducted using ANOVA and Duncan’s test in SPSS Statistics 22 (IBM, Armonk, NY, USA); differences were considered significant if *p* < 0.05.

## 3. Results

### 3.1. Textural Quality Characteristics Analysis

Table 1 illustrates the effects of different extrusion parameters on the quality characteristics (textural properties, expansion ratio, and bulk density) of the PMP.

#### 3.1.1. MP Addition Amount

Table 1 shows that increasing the amount of MP added generally decreased the hardness of the PMP (*p* < 0.05). This trend can be attributed to the higher moisture content in MP, leading to a less dense structure in the PMP after extrusion. Excessive moisture can dilute the protein, reduce protein cross-linking sites, and adversely affect protein aggregation and network structure formation [36]. While the hardness decreased, the gumminess and chewiness showed an increase, indicating opposing changes in the texture parameters. Consequently, the addition of excessive MP resulted in a sticky extrudate that lacked springiness. Similar results were reported in the study by Zang et al. [37], which may be due to the interaction between molecules, allowing PMP to retain more water. The texturization degree directly reflects the fiber structure characteristics of PMP. Within the texturization degree range of 1.24 to 1.65, the fiber structure density was observed to increase, as shown in Table 1. From Table 1, the expansion ratio of the PMP showed a decreasing trend as the MP addition amount increased. However, the trends of the bulk density and texturization degree were similar, both showing an increase first and then a decrease. This may be because the increase in the water content due to the MP makes the PMP more compact.

#### 3.1.2. Screw Speed

Screw speed is a key parameter that significantly affects the textural properties of plant protein. As shown in Table 1, as the screw speed gradually increased from 140 rpm to 220 rpm, all the PMPR texture parameters showed a typical unimodal distribution. Hardness, adhesiveness, and chewiness reached their peak values simultaneously at 180 rpm, corresponding to 1780 g, 1478, and 1004, respectively. This is likely because the increased melt viscosity at this speed enhances the texturing effect of vegetable protein [38]. The expansion ratio also reached its maximum within this range (1.65). However, further increases or decreases in screw speed led to a significant reduction in these parameters, accompanied by an increase in bulk density. The product transitioned rapidly from a dense and elastic state to a loose and fragile one. These findings indicated that 180 rpm represents a critical inflection point in texture formation, and deviations from this speed resulted in the deterioration of the texturized quality. When it reached a certain level, excessively high screw speeds may destroy the texturization degree, which is similar to the findings of the study by Ferawati et al. [39].

#### 3.1.3. Extrusion Temperature

Extrusion temperature is another key parameter in processing plant protein. Using the appropriate extrusion temperature not only improves the structural and functional properties of plant protein, but also significantly affects its textural properties [40]. The results from the table show that increasing the extrusion temperature increased the hardness of the PMP. However, excessively high extrusion temperatures led to overly high hardness values, which was detrimental to texture properties. The gumminess of the PMP increased, while the chewiness first rose and then decreased. Similarly, the bulk density of the PMP also changed in a similar manner, but its expansion ratio decreased slightly, though not significantly (*p* ˃ 0.05). During the extrusion process, thermal treatment promotes the alignment and re-crosslinking of protein molecular chains, forming a fibrous structure. The formation of this structure relies on precise temperature control to ensure appropriate thermal denaturation and restructuring of the proteins [41].

### 3.2. Color Analysis

Food color is a crucial indicator for determining food acceptance [42]. Table 2 presents the effects of different extrusion parameters on the color of the PMP.

#### 3.2.1. MP Addition Amount

As the amount of MP increased, the moisture content of the extruded protein rose, resulting in an increase in the L* value, a gradual decrease in the a* value, and an initial increase followed by a decrease in the b* value (*p* < 0.05). These changes are likely related to the higher moisture content in the mixed materials. The addition of moisture can enhance the color of the brushed protein, consistent with the findings of Barnés-Calle et al. [43].

#### 3.2.2. Screw Speed

Increasing the screw speed led to a gradual increase in brightness, with the a* value first increasing and then decreasing, and the b* value exhibiting similar changes (*p* < 0.05). This may be due to the increased screw speed and the reduced residence time of the material during extrusion. Under consistent temperature and moisture conditions, a shorter extrusion time resulted in insufficient melting of the material.

#### 3.2.3. Extrusion Temperature

Additionally, a rise in the extrusion temperature significantly reduced the L* of the PMP due to the Maillard reaction (*p* < 0.05). This is consistent with the findings of Yu et al. [44], who observed that under high-temperature conditions, reducing sugars in cornstarch and free amino acids in fish protein undergo the Maillard reaction, producing a small amount of brown substances. For plant-based proteins, lighter hues (higher L* values) are often associated with freshness and reduced processing artifacts. An increased L* value and decreased a* value indicate brightening and a reduced red/green tone and may be in line with consumers’ preference for the appearance of natural, minimal processing. Optimal extrusion parameters should preserve natural color profiles to align with clean-label trends.

### 3.3. Oil- and Water-Holding Capacities (OHC and WHC)

**Figure 1:**
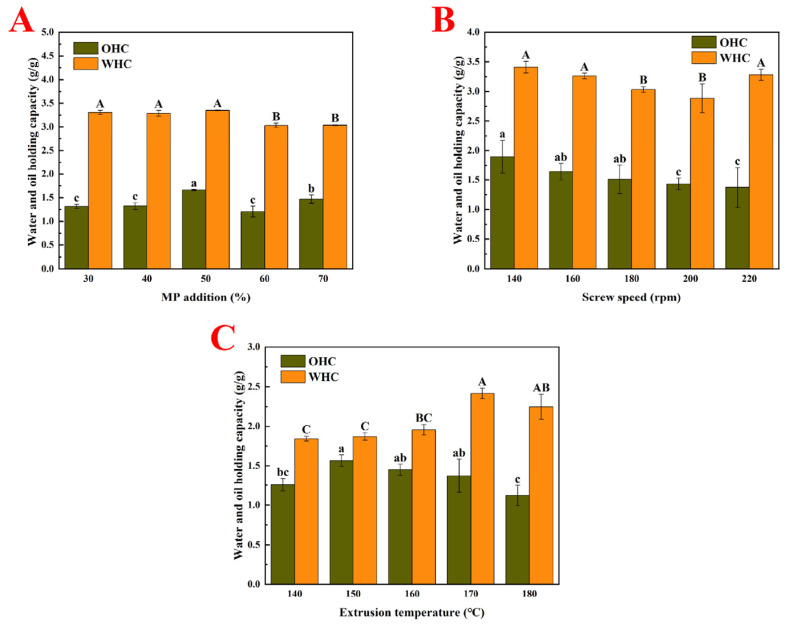
Water- and oil-holding capacities of PMP produced using different extrusion parameters. (**A**). Effect of different MP addition amounts on water- and oil-holding capacities of PMP. (**B**). Effect of different screw speeds on water- and oil-holding capacities of PMP. (**C**). Effect of different extrusion temperatures on water- and oil-holding capacities of PMP. Capital letters indicate significant differences among WHC, while lowercase letters indicate significant differences among OHC.

As shown in Figure 1, the amount of MP added, along with the screw speed and extrusion temperature, significantly affected the oil-holding capacity (OHC) and water-holding capacity (WHC) of the PMP.

#### 3.3.1. MP Addition Amount

In Figure 1A, when the amount of MP was 50%, the PMP exhibited the highest OHC and WHC of 1.66 g/g and 3.35 g/g, respectively. This may be because, with a 50% addition, the protein network achieved its optimal state, providing the maximum OHC and WHC. Increasing the MP amount resulted in a decrease in the total pea protein content, which affected the OHC/WHC due to changes in protein interaction dynamics. Meanwhile, beyond this optimal addition level, excessive water may result in an overly tight network structure or weakened interactions between proteins, thereby reducing the oil- and water-holding capacities [45,46].

#### 3.3.2. Screw Speed

Figure 1B shows that different screw speeds had a significant impact on the OHC and WHC of the PMP. As the screw speed increased, the OHC gradually decreased, while the WHC of the PMP initially decreased and then increased. Screw speed influences the mixing and shear force. An appropriate shear force helps in creating a uniform dispersion of proteins and other ingredients, thereby improving the WHC. However, excessively high rotational speeds may damage the protein structure and affect the network formation ability, and thus reduce the WHC. Additionally, increased screw speed may also cause more frictional heat, affecting the thermal stability and OHC and WHC of the protein.

#### 3.3.3. Extrusion Temperature

Figure 1C shows that as the extrusion temperature increased, both the OHC and WHC of the PMP first increased and then decreased. Specifically, when the extrusion temperature was 150 °C, the OHC of the PMP reached its maximum; when the temperature rose to 170 °C, its WHC reached its peak. This phenomenon may be related to changes in the protein structure caused by high-temperature treatment, exposure or changes to surface hydrophobic/hydrophilic groups, or changes in the aggregation state. Under the influence of high pressure inside the extruder, proteins may undergo excessive denaturation or thermal damage, which disrupts their structure and reduces their OHC and WHC. Higher temperatures promote protein unfolding, exposing more hydrophobic and hydrophilic groups, which facilitate the physical retention and binding of oil [41]. These findings aligned with the SEM observations, which revealed fibrous and highly cross-linked structures resulting from the aggregation of protein molecules (PPI and MP) [13].

### 3.4. Determination of Sulfhydryl (SH) Group and Disulfide Bond (DB) Contents

Table 3 shows the effects of the different extrusion parameters on the SH and DB contents of the PMP.

#### 3.4.1. MP Addition Amount

The amount of added MP, screw speed, and extrusion temperature significantly influenced the free sulfhydryl (FSH) group, total sulfhydryl (TSH) group, and DB contents in the PMP (*p* < 0.05). Changes in FSH and TSH moieties result from the formation of new inter- and intramolecular cross-links following cysteine oxidation or cystine reduction, which are attributed to the rearrangement of intermolecular disulfide cross-links during processing [47]. During the extrusion process, the oxidation of SH groups forms DBs, thereby stabilizing the structure and functional properties of tissue proteins. As shown in Table 3, increasing the amount of MP initially raised and then lowered the disulfide bond content. Notably, a higher addition level (50%) enhanced the TSH and DB contents. However, when the MP addition amount reached 70%, although the FSH content increased, the TSH and DB contents decreased, indicating that excessive MP addition may impair protein structure and functionality.

#### 3.4.2. Screw Speed

With increasing screw speed, the DB content in the PMP initially showed a trend of first increasing and then decreasing. This may be because higher screw speeds during extrusion generate greater shear forces and heat, which increases the DB content. However, excessively high screw speeds reduced the extrusion time, affecting protein cross-linking and causing data fluctuations.

#### 3.4.3. Extrusion Temperature

Extrusion temperature had a minimal impact on the FSH content but significantly affected the TSH and DB contents (*p* < 0.05). As the extrusion temperature increased, the FSH content showed an upward trend, while the TSH content first rose and then decreased, similar to the DB content, indicating that high-temperature conditions are favorable for DB formation. When the extrusion temperature was below 160 °C, protein conformational unfolding exposed the originally buried thiols, leading to simultaneous increases in the FSH and TSH contents. However, especially at an extrusion temperature of 160 °C, the exposed FSH groups rapidly converted to DBs, significantly increasing the DB content, which may be related to enhanced inter- or intramolecular DB cross-linking induced by high temperatures. A higher extrusion temperature (160 °C) may promote the formation of disulfide bonds. B. Zhang et al. [48] reported that the appropriate temperature during extrusion causes small conformational changes in pea proteins, resulting in an increase in the DB content.

### 3.5. Surface Hydrophobicity (H_0_)

**Figure 2:**
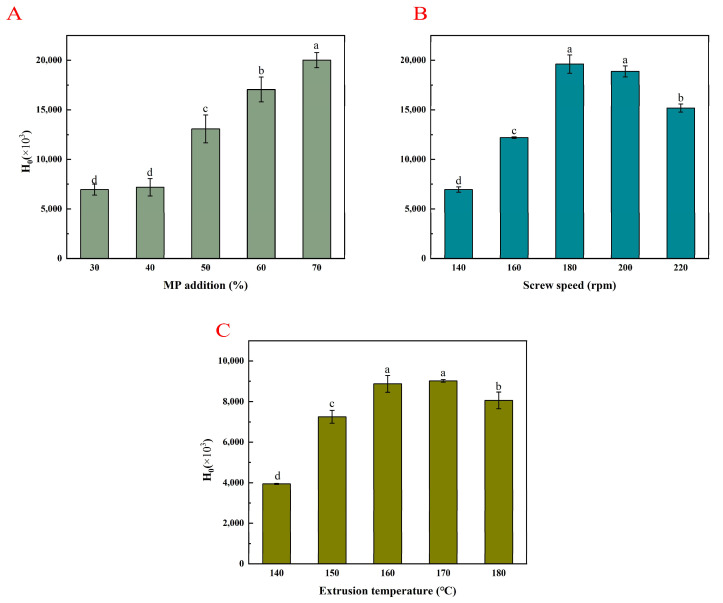
Surface hydrophobicity of PMP produced using different extrusion parameters. (**A**). Effect of different MP addition amounts on surface hydrophobicity of PMP. (**B**). Effect of different screw speeds on surface hydrophobicity of PMP. (**C**). Effect of different extrusion temperatures on surface hydrophobicity of PMP. Lowercase letters indicate significant differences among samples.

Figure 2 shows the effect of the different extrusion parameters on the surface hydrophobicity of the PMP.

#### 3.5.1. MP Addition Amount

As shown in Figure 2A, the surface hydrophobicity of the PMP gradually increased with the amount of MP added. This may be due to the interactions between protein molecules and protein aggregation in the extruder covering the hydrophobic sites on the protein surface, thereby inhibiting their binding with ANS. Additionally, the increased moisture content during extrusion effectively enhanced the surface hydrophobicity, causing protein denaturation and exposing more hydrophobic regions, making the protein structure more flexible [49].

#### 3.5.2. Screw Speed

As shown in Figure 2B, as the screw speed increased, the surface hydrophobicity of the protein first increased and the decreased. A low speed may produce an insufficient shear force, making it difficult for proteins to fully unfold, resulting in limited exposure of hydrophobic groups and lower surface hydrophobicity [50]. An appropriate screw speed enhances the shear force, promoting protein dissociation and rearrangement, reducing β-folding, and increasing the ratio of β-turns and α-helices, making hydrophobic regions more exposed. This was consistent with the subsequent results from the protein secondary structure analysis. Higher screw speeds and temperatures allowed the protein molecular chains to unfold more easily and exposing more active groups, promoting protein–protein interactions and protein–water interactions. The formation of more protein aggregates covered the hydrophobic sites on the protein surface and inhibited the binding to ANS [23], resulting in a decrease in surface hydrophobicity.

#### 3.5.3. Extrusion Temperature

As shown in Figure 2C, as the extrusion temperature increased, the surface hydrophobicity of the protein first increased and then decreased. An appropriate increase in the extrusion temperature disrupts the secondary and tertiary structures of proteins, promoting the exposure of buried hydrophobic groups [51] and increasing surface hydrophobicity. However, higher extrusion temperatures bring more heat, intensifying the Maillard reaction, which affects the coverage of hydrophilic groups on the surface of Maillard reaction products, inhibiting the binding to ANS, leading to a decrease in surface hydrophobicity.

### 3.6. Scanning Electron Microscopy (SEM) Observations

**Figure 3:**
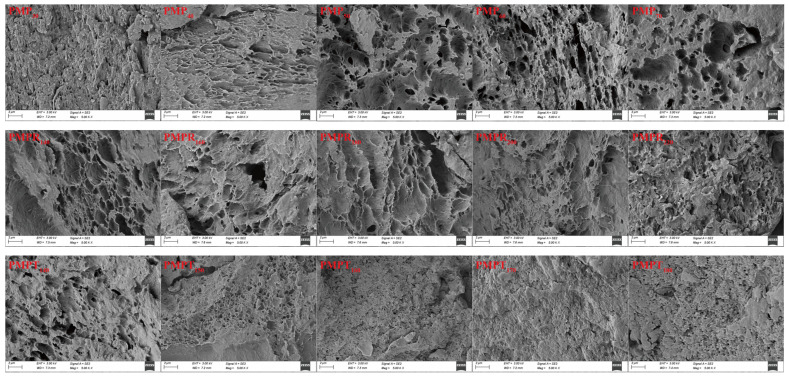
Scanning electron microscopy (SEM) images of effects of the different extrusion parameters on the PMP structure. Note: The subscript number after PMP represents the amount of MP added; the subscript number after PMPR represents the screw speed; the subscript number after PMPT represents the extrusion temperature. PMP50: control for the MP series; PMPR180: control for the screw speed series; PMPT140: control for the processing temperature series. Please note that each control sample was independently batch-produced.

Figure 3 shows SEM images of the effect of the different extrusion parameters on the PMP structure. The SEM images indicated that changes in the extrusion parameters significantly affected the PMP.

#### 3.6.1. MP Addition Amount

The protein structure was affected by the MP content. When the MP content was 30%, the protein surface was rough but exhibited evenly distributed gaps. As the MP content increased, the gaps became larger and their distribution became less uniform. At an MP content of 50%, the gaps were evenly distributed and larger.

#### 3.6.2. Screw Speed

The screw speed also significantly influenced the PMP structure. As shown in Figure 3, the network structure of the pea protein and MP was pronounced at a low screw speed of 140 rpm. As the screw speed increased, the shear force intensified, resulting in PMP with a more balanced surface. Shearing forces made the protein appear more organized. However, due to the reduced interaction time between proteins at higher screw speeds, the network structure gradually becomes less organized.

#### 3.6.3. Extrusion Temperature

The extrusion temperature had a marked effect on the PMP structure as well. At a lower temperature of 140 °C, the protein in the PMP exhibited gaps of varying sizes. With increasing temperature, the gaps narrowed and the protein surface became smoother, likely due to high temperatures causing polymerization. This result may be due to large protein molecules breaking down into smaller ones, which filled the gaps.

### 3.7. Correlation Analysis to Statistically Link the Textural Properties, Color Values, and Sulfhydryl/Disulfide Bond Data

As shown in Figure 3, this study comprehensively examined the impact of varying high-moisture extrusion parameters on the textural development of PMP and the associated physicochemical properties.

#### 3.7.1. MP Addition Amount

**Figure 4:**
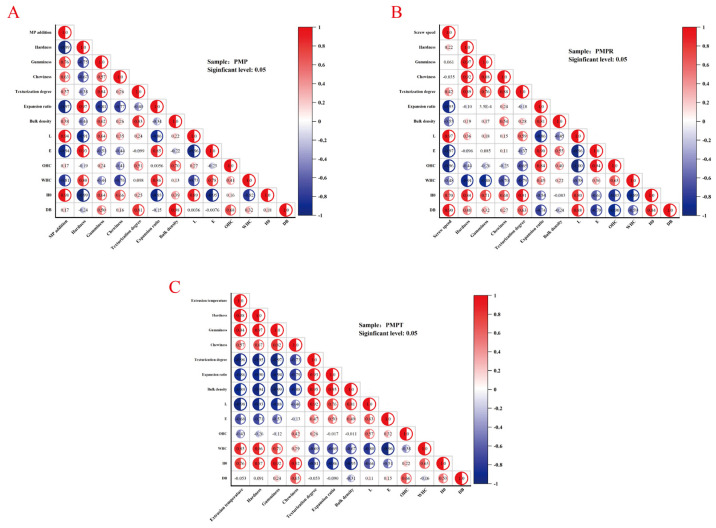
Effect of different process parameters on texturization degree and related indicators. (**A**). MP addition amount. (**B**). Screw speed. (**C**). Extrusion temperature.

Figure 4A shows the influence of the MP addition amount on the texturization degree and its related attributes. An elevated MP content markedly intensified disulfide bond cross-linking, fostering the formation of a dense protein surface, thereby yielding robust positive correlations between texturization degree and gumminess, chewiness, bulk density, and disulfide bond content. Conversely, the expansion ratio decreased and hardness diminished under higher MP addition amounts.

#### 3.7.2. Screw Speed

Figure 4B shows the role of screw speed in modulating texturization. An increased screw speed facilitated preferential protein molecular alignment, exposure of hydrophobic domains, and disulfide bond formation through pronounced shear forces, significantly enhancing both the texturization degree and textural properties such as hardness and chewiness. However, the densely packed microstructure induced by high shear conditions compromised the OHC and WHC.

#### 3.7.3. Extrusion Temperature

Figure 4C illustrates the effect of extrusion temperature on texturization. Higher temperatures augmented the protein melt plasticity expansion ratio, resulting in strong positive associations between texturization degree and expansion ratio, L*, and bulk density. Nevertheless, excessive thermal inputs prompted protein over-aggregation and network deterioration, consequently diminishing the hardness, gumminess, and surface hydrophobicity.

### 3.8. Fourier Transform Infrared Spectroscopy (FTIR)

**Figure 5:**
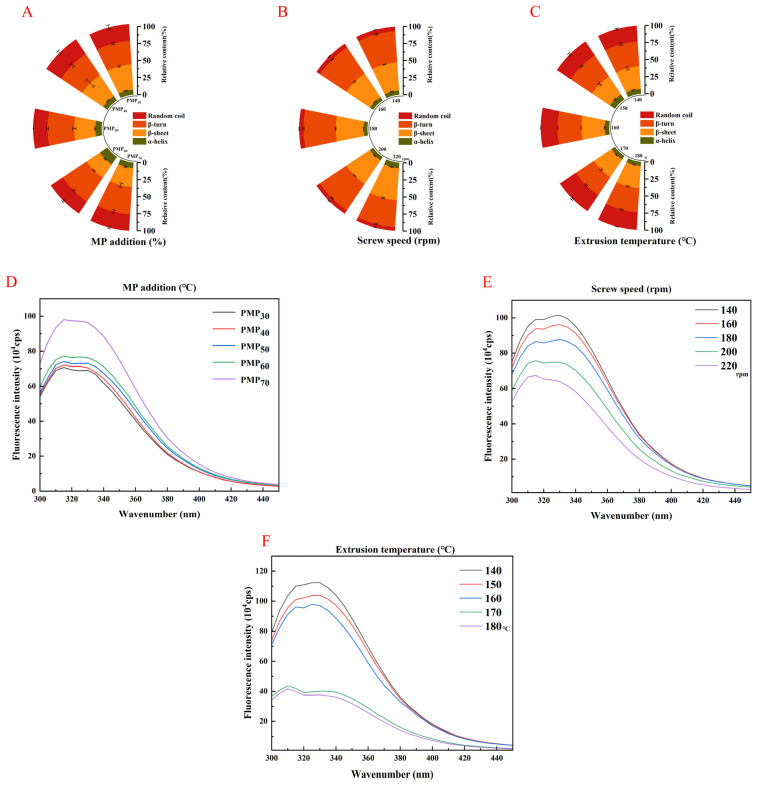
Protein structure of PMP produced using different extrusion parameters. (**A**). Effect of different MP addition amounts on infrared secondary protein structure of PMP. (**B**). Effect of different screw speeds on infrared secondary protein structure of PMP. (**C**). Effect of different extrusion temperatures on infrared secondary protein structure of PMP. (**D**). Effect of different MP addition amounts on intrinsic tryptophan fluorescence of PMP. (**E**). Effect of different screw speeds on intrinsic tryptophan fluorescence of PMP. (**F**). Effect of different extrusion temperatures on intrinsic tryptophan fluorescence of PMP.

Figure 5 shows the effect of the different extrusion parameters on the secondary structure of the PMP. The amide I band in different regions represents specific protein secondary structures: α-helices (1646–1664 cm^−1^), β-sheets (1615–1637 cm^−1^ and 1682–1700 cm^−1^), β-turns (1664–1681 cm^−1^), and random coils (1637–1645 cm^−1^) [52]. As the amount of MP added increased and the moisture content increased, the β-sheet content first increased and then decreased; the random coil and β-turn contents first decreased and then increased; and the α-helix content did not change significantly. This may be because α-helices are stable structures that are not easily changed, which is similar to the results of Li et al. [53].

Moreover, the sum of the intramolecular β-sheet and α-helix contents is called the total intramolecular hydrogen bonding interactions, which represents the tightness of the protein [54]. α-helices and β-sheets are ordered structures, while β-turns and random coil structures are loose and disordered structures [55]. Therefore, an increase in moisture content can enhance intramolecular hydrogen bonds and increase the β-sheet content, which was beneficial to the formation of fibrous structures in the PMP. However, an excessive moisture content resulted in a decrease in the proportion of β-sheets. This may have been due to protein entanglement and altered filling properties [56], which caused the hydrogen bonds within the protein to break and converted the β-sheets into random coils and β-turns [57]. The covalent binding of pea protein and mung bean protein can enhance the interaction between protein molecules [58]. In addition, as the extrusion temperature and screw speed increased, the β-sheet content gradually increased and the random coil and β-turn contents gradually decreased while the percentage change in α-helical structures was not obvious. Changes in the β-sheet ratio may be related to protein aggregation. In thermally denatured proteins, the increase in β-sheet structure is caused by protein aggregation during heating, resulting in a more rigid structure [42]. The decrease in the ratio of random coils and β-turns indicates that increasing the extrusion temperature and screw speed promotes the transformation of random coil and β-turn structures into β-sheets. The energy generated by the extrusion process exceeds the formation of hydrogen bonds, causing the required activation energy to be exceeded.

### 3.9. Intrinsic Tryptophan Fluorescence 

Intrinsic fluorescence was used to characterize the spatial conformational changes of proteins, and the results are shown in Figure 5. The fluorescence intensity gradually increased with an increase in the amount of MP added and decreased with an increase in screw speed and extrusion temperature. In natural proteins, most fluorescent chromophore residues are located within the protein molecules. The denaturation of proteins after extrusion gradually exposes the side chains of the fluorescent chromophore residues, thereby reducing the fluorescence intensity within the protein molecules. This is caused by increasing intermolecular hydrophobic interactions and protein–protein aggregation. The harsh conditions at high extrusion temperatures and screw speeds caused the protein to be highly denatured, thereby increasing the exposure of tryptophan residues and changing the microenvironment of the fluorescent chromophore. This ultimately led to a weakening of the fluorescence intensity within the protein molecule [40]. The high thermal energy input and mechanical damage that occurred in the extruder caused protein denaturation, which destroyed hydrogen bonds, hydrophobic bonds, and other interaction forces that maintained the proteins’ spatial structure. This led to an increased exposure of tryptophan residues to aqueous solutions, resulting in a steady increase in the polarity of their environment.

### 3.10. Three-Dimensional Response Surface Model for Effects of Different Extrusion Parameters on Response Value

**Figure 6:**
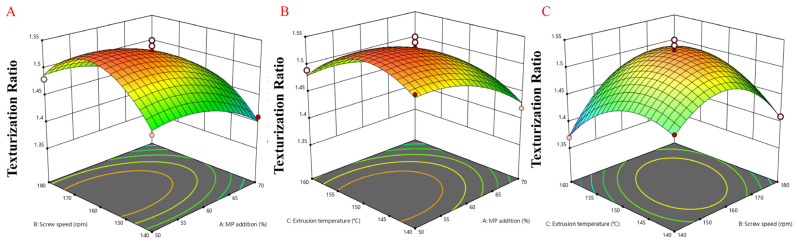
Three-dimensional response surface modeling graph for response variables. (**A**). Three-dimensional response surface plot of texturization degree of PMP as a function of MP addition amount (%) and screw speed (rpm). (**B**). Three-dimensional response surface plot of texturization degree of PMP as a function of MP addition amount (%) and extrusion temperature (°C). (**C**). Three-dimensional response surface plot of texturization degree of PMP as a function of screw speed (rpm) and extrusion temperature (°C).

Products with a high degree of organization have a stronger fiber structure. On the contrary, a lower degree of organization will lead to lower product quality. Therefore, three factor levels were selected based on the experimental results. Interactions between variables were visualized and optimal conditions were identified to maximize the texturization degree, which is a key quality metric that is linked to consumer acceptance. The 3-dimensional response surface modeling graph for the response variables is shown in Figure 6, which shows the relationship between the independent factors and response factor. This graph was generated by varying two independent variables to ascertain the response variables. The texturization degree was one of the key indicators for evaluating the quality of the PMP. Its level is directly related to the texture and taste of PMP, which in turn affects consumer acceptance. The experiments and analyses, as presented in Table 4, revealed that different extrusion parameters significantly impacted the texturization degree of the PMP (*p* < 0.05). This finding shows that the product quality of PMP can be significantly improved by optimizing the extrusion parameters. Through calculation and simulation, the optimal parameter combination to achieve the highest texturization degree of the PMP was determined. Specifically, when the MP addition amount was precisely controlled at 53%, the screw speed was set at 160 rpm, and the extrusion temperature was maintained at 150 °C, the texturization degree of the PMP reached the theoretical maximum value of 1.55 (*p* < 0.05). To validate the results of the response surface methodology, additional experiments were performed to confirm whether the optimal parameter combination could consistently achieve the theoretical maximum texturization degree. The experimental conditions were 53% MP, a screw speed of 160 rpm, and an extrusion temperature of 150 °C. In repeated trials, the measured texturization degree values were very close to the theoretical prediction, with an average of 1.53 and a standard deviation of 0.02. This suggests that the selected experimental parameters are capable of reliably achieving a texturization degree near the theoretical maximum.

## 4. Conclusions

This study introduces a homemade mung bean protein extract solution as a water replacement in HME for the first time. Single-factor experiments examined the effects of MP addition amount, screw speed, and temperature on the quality and structure of a pea–mung bean composite (PMP), and RSM was used to optimize texturization. A MP content of 50–70% increased surface hydrophobicity, disulfide bonds, ordered secondary structures, and fluorescence intensity, and significantly improved the WHC, OHC, bulk density, and texturization. Screw speeds of 160–180 rpm improved the PMP quality via higher shear and a shorter residence time, whereas higher temperatures darkened the color and reduced the texturization and bulk density (*p* < 0.05). The optimal conditions were 53% MP, 160 rpm, and 150 °C, yielding a texturization degree of 1.55. Future work should incorporate sensory evaluation and instrumental texture analysis to link structural improvements to consumer acceptance for meat analog applications.

## Figures and Tables

**Table 1:** Effect of different extrusion parameters on the quality characteristics of PMP.

Sample	Hardness/g	Gumminess/g∙sec	Chewiness	Texturization Degree	Expansion Ratio	Bulk Density/g∙cm^3^
PMP_30_	1805.33 ± 9.50 ^a^	1376.33 ± 18.93 ^b^	984.67 ± 11.85 ^c^	1.24 ± 0.07 ^d^	1.057 ± 0.014 ^a^	2.00 ± 0.23 ^c^
PMP_40_	1796.33 ± 24.58 ^bc^	1467.33 ± 18.50 ^a^	995.67 ± 9.29 ^bc^	1.53 ± 0.01 ^b^	1.047 ± 0.010 ^ab^	2.17 ± 0.04 ^bc^
PMP_50_	1780.00 ± 19.92 ^abc^	1478.33 ± 17.10 ^a^	1004.33 ± 19.50 ^bc^	1.65 ± 0.02 ^a^	1.044 ± 0.018 ^ab^	3.00 ± 0.27 ^a^
PMP_60_	1768.33 ± 9.45 ^bc^	1484.67 ± 8.08 ^a^	1076.33 ± 15.31 ^a^	1.50 ± 0.05 ^bc^	1.032 ± 0.009 ^b^	2.47 ± 0.09 ^b^
PMP_70_	1759.67 ± 8.39 ^c^	1476.33 ± 14.98 ^a^	1016.33 ± 14.19 ^b^	1.43 ± 0.04 ^c^	1.031 ± 0.008 ^b^	2.31 ± 0.11 ^bc^
PMPR_140_	1058.00 ± 11.79 ^f^	993.33 ± 10.02 ^c^	885.67 ± 14.15 ^c^	1.22 ± 0.15 ^b^	1.047 ± 0.006 ^a^	2.79 ± 0.15 ^ab^
PMPR_160_	1270.00 ± 15.62 ^c^	1069.67 ± 14.50 ^b^	935.33 ± 17.95 ^b^	1.45 ± 0.11 ^ab^	1.045 ± 0.012 ^a^	2.82 ± 0.14 ^ab^
PMPR_180_	1780.00 ± 19.92 ^a^	1478.33 ± 17.10 ^a^	1004.33 ± 19.50 ^a^	1.65 ± 0.02 ^a^	1.044 ± 0.018 ^a^	3.00 ± 0.06 ^a^
PMPR_200_	1674.67 ± 14.57 ^b^	1460.00 ± 13.75 ^a^	944.67 ± 20.23 ^b^	1.51 ± 0.04 ^a^	1.033 ± 0.010 ^a^	2.51 ± 0.27 ^b^
PMPR_220_	1092.67 ± 16.29 ^d^	852.67 ± 8.39 ^d^	875.33 ± 18.50 ^c^	1.40 ± 0.19 ^ab^	1.032 ± 0.011 ^a^	2.61 ± 0.11 ^b^
PMPT_140_	1780.00 ± 19.92 ^a^	1478.33 ± 17.10 ^c^	1004.33 ± 19.50 ^c^	1.65 ± 0.02 ^a^	1.044 ± 0.018 ^a^	3.00 ± 0.27 ^a^
PMPT_150_	1850.33 ± 22.50 ^c^	1524.67 ± 22.28 ^b^	1099.67 ± 24.09 ^ab^	1.56 ± 0.02 ^b^	1.038 ± 0.007 ^a^	2.65 ± 0.14 ^b^
PMPT_160_	1899.67 ± 17.50 ^b^	1548.33 ± 9.07 ^ab^	1124.33 ± 22.50 ^a^	1.53 ± 0.03 ^b^	1.039 ± 0.012 ^a^	2.53 ± 0.06 ^b^
PMPT_170_	1953.33 ± 19.86 ^a^	1552.33 ± 11.85 ^ab^	1088.67 ± 8.14 ^b^	1.52 ± 0.04 ^b^	1.037 ± 0.004 ^a^	2.51 ± 0.06 ^b^

Different letters in the same column indicate a significant difference at *p* < 0.05 in the specific subgroup. Note: The number after PMP represents the amount of MP added; the number after PMPR represents the screw speed; the number after PMPT represents the extrusion temperature. PMP50: control for the MP series; PMPR180: control for the screw speed series; PMPT140: control for the processing temperature series. Please note that each control sample was independently batch-produced.

**Table 2:** Effect of different extrusion parameters on the color of PMP.

Sample	L*	a*	b*	Δ*E*
PMP_30_	78.64 ± 0.21 ^a^	4.84 ± 0.18 ^a^	23.70 ± 0.23 ^b^	30.47 ± 0.26 ^a^
PMP_40_	79.42 ± 0.52 ^a^	4.68 ± 0.15 ^a^	24.48 ± 0.44 ^a^	30.54 ± 0.54 ^a^
PMP_50_	79.78 ± 0.50 ^b^	4.32 ± 0.31 ^b^	23.90 ± 0.30 ^b^	29.81 ± 0.31 ^b^
PMP_60_	80.02 ± 0.22 ^c^	3.70 ± 0.19 ^c^	23.76 ± 0.22 ^b^	29.44 ± 0.20 ^b^
PMP_70_	81.76 ± 0.60 ^d^	2.90 ± 0.31 ^d^	23.70 ± 0.32 ^b^	28.24 ± 0.46 ^c^
PMPR_140_	77.34 ± 0.68 ^c^	1.48 ± 0.48 ^c^	19.76 ± 0.36 ^b^	28.11 ± 0.77 ^a^
PMPR_160_	78.44 ± 0.42 ^b^	1.76 ± 0.34 ^bc^	20.00 ± 0.37 ^ab^	27.50 ± 0.41 ^ab^
PMPR_180_	78.84 ± 0.58 ^ab^	1.84 ± 0.18 ^abc^	20.38 ± 0.34 ^a^	27.48 ± 0.50 ^ab^
PMPR_200_	79.26 ± 0.80 ^a^	2.22 ± 0.26 ^a^	20.22 ± 0.49 ^ab^	27.13 ± 0.37 ^bc^
PMPR_220_	79.62 ± 0.28 ^a^	1.94 ± 0.22 ^ab^	19.94 ± 0.38 ^ab^	26.64 ± 0.47 ^c^
PMPT_140_	79.90 ± 0.37 ^a^	2.66 ± 0.64 ^b^	23.18 ± 0.33 ^a^	28.93 ± 0.37 ^a^
PMPT_150_	79.72 ± 0.22 ^a^	4.12 ± 0.87 ^a^	23.02 ± 0.39 ^a^	29.15 ± 0.39 ^a^
PMPT_160_	78.68 ± 0.59 ^b^	1.88 ± 0.43 ^c^	22.14 ± 0.65 ^b^	28.86 ± 0.72 ^a^
PMPT_170_	78.24 ± 0.61 ^c^	3.90 ± 0.16 ^a^	18.62 ± 0.50 ^c^	27.03 ± 0.71 ^c^
PMPT_180_	77.20 ± 0.54 ^d^	4.32 ± 0.33 ^a^	19.02 ± 0.24 ^c^	28.14 ± 0.40 ^b^

Different letters in the same column indicate a significant difference at *p* < 0.05 in the specific subgroups. Note: The subscript number after PMP represents the amount of MP added; the subscript number after PMPR represents the screw speed; the subscript number after PMPT represents the extrusion temperature. PMP50: control for the MP series; PMPR180: control for the screw speed series; PMPT140: control for the processing temperature series. Please note that each control sample was independently batch-produced.

**Table 3:** Effect of different extrusion parameters on SH and DB contents of PMP.

Sample	FSH (μmol/g)	TSH (μmol/g)	DB (μmol/g)
PMP_30_	11.44 ± 0.21 ^b^	45.02 ± 0.62 ^d^	16.77 ± 0.20 ^d^
PMP_40_	11.48 ± 0.07 ^b^	46.73 ± 0.75 ^c^	17.62 ± 0.34 ^c^
PMP_50_	11.56 ± 0.25 ^b^	55.96 ± 0.37 ^a^	22.20 ± 0.06 ^a^
PMP_60_	11.73 ± 0.19 ^b^	49.42 ± 0.79 ^b^	18.85 ± 0.30 ^b^
PMP_70_	12.87 ± 0.61 ^a^	47.54 ± 0.64 ^c^	17.34 ± 0.02 ^cd^
PMPR_140_	22.11 ± 0.50 ^d^	45.83 ± 0.49 ^a^	11.86 ± 0.04 ^a^
PMPR_160_	23.91 ± 0.47 ^c^	48.20 ± 0.37 ^c^	12.15 ± 0.38 ^a^
PMPR_180_	27.73 ± 0.74 ^a^	58.25 ± 0.75 ^a^	15.26 ± 0.34 ^bc^
PMPR_200_	26.50 ± 0.55 ^b^	56.13 ± 0.74 ^b^	14.82 ± 0.55 ^c^
PMPR_220_	24.15 ± 0.62 ^c^	55.47 ± 0.74 ^b^	15.66 ± 0.14 ^a^
PMPT_140_	20.40 ± 0.40 ^b^	38.06 ± 0.66 ^d^	8.83 ± 0.23 ^bc^
PMPT_150_	21.22 ± 0.29 ^b^	40.77 ± 0.69 ^c^	9.77 ± 0.47 ^b^
PMPT_160_	22.29 ± 0.63 ^a^	45.55 ± 0.71 ^a^	11.62 ± 0.66 ^a^
PMPT_170_	22.53 ± 0.33 ^a^	42.12 ± 0.28 ^b^	9.79 ± 0.30 ^b^
PMPT_180_	23.02 ± 0.74 ^a^	40.26 ± 0.77 ^c^	8.62 ± 0.71 ^c^

Different letters in the same column indicate a significant difference at *p* < 0.05 in the specific subgroups. Note: FSH, sulfhydryl; TSH, total sulfhydryl; DB, disulfide bond. The subscript number after PMP represents the amount of MP added; the subscript number after PMPR represents the screw speed; the subscript number after PMPT represents the extrusion temperature. PMP50: control for the MP series; PMPR180: control for the screw speed series; PMPT140: control for the processing temperature series. Please note that each control sample was independently batch-produced.

**Table 4:** Experimental design and results for extruder operating conditions.

No.	MP Addition Amount (%)	Screw Speed (rpm)	Extrusion Temperature (°C)	Texturization Degree
1	50	140	150	1.45
2	70	140	150	1.41
3	50	180	150	1.48
4	70	180	150	1.42
5	50	160	140	1.51
6	70	160	140	1.42
7	50	160	160	1.49
8	70	160	160	1.42
9	60	140	140	1.45
10	60	180	140	1.41
11	60	140	160	1.37
12	60	180	160	1.45
13	60	160	150	1.53
14	60	160	150	1.52
15	60	160	150	1.55
16	60	160	150	1.51
17	60	160	150	1.54
Source of variance	Texturization degree		Dependent variable (Y)	Equation
	F	*p*		
Quadratic model	23.48	0.0002	Texturization degree	Y = 1.53 − 0.0325A + 0.01B − 0.0075C − 0.005AB + 0.005AC + 0.03BC − 0.025A^2^ − 0.065B^2^ − 0.045C^2^
MP addition (A)	39.43	0.0004		
Screw speed (B)	3.73	0.0946		
Extrusion temperature (C)	2.1	0.1906		
AB	0.4667	0.5165		
AC	0.4667	0.5165		
BC	16.8	0.0046		
A^2^	12.28	0.0099		
B^2^	83.02	<0.0001		
C^2^	39.79	0.0004		
R^2^	0.97			
R_adj_^2^	0.93			

## Data Availability

The original contributions presented in this study are included in the article. Further inquiries can be directed to the corresponding author.
